# Surgical treatment for gastrointestinal neuroendocrine tumors

**DOI:** 10.1002/ags3.12396

**Published:** 2020-09-12

**Authors:** Kojiro Eto, Naoya Yoshida, Shiro Iwagami, Masaaki Iwatsuki, Hideo Baba

**Affiliations:** ^1^ Department of Gastroenterological Surgery Graduate School of Medical Sciences Kumamoto University Kumamoto Japan

**Keywords:** cytoreductive surgery, endoscopic resection, enterectomy with lymph node dissection, gastrointestinal neuroendocrine tumors

## Abstract

Neuroendocrine tumors (NETs) are rare neoplasms, with an estimated annual incidence of 6.9/100 000. They arise from cells of the diffuse endocrine system, which are mainly dispersed throughout the gastrointestinal (GI), pancreatic, and respiratory tracts. The incidence of GI‐NETs has recently begun to show a steady increase. According to the Surveillance, Epidemiology, and End Results database, 53% of patients with NETs present with localized disease, 20% with locoregional disease, and 27% with distant metastases at the time of diagnosis. Surgery is the mainstay for the treatment of locoregional GI‐NETs. Endoscopic resection is an option for well‐differentiated early GI‐NETs, which are thought to very rarely metastasize to lymph nodes. A lesion that is technically difficult to resect via endoscopy is an indication for local resection (partial resection without lymph node dissection). GI‐NETs with possible lymph node metastasis is an indication for enterectomy with lymph node dissection. For NETs with metastatic lesions, cytoreduction surgery can control hormonal hypersecretion and alleviate symptoms; therefore, cytoreduction surgery is recommended. The indications for surgery vary and are based on the organ where the NET arose; therefore, an understanding of the patient's clinical state and individualized treatment that is based on the characteristics of the patient's GI‐NET is needed. This review summarizes surgical treatments of GI‐NETs in each organ.

## Graphical abstract

The indications for surgery vary and are based on the organ where the neuroendocrine tumors (NET) arose; therefore, an understanding of the patient's clinical state and individualized treatment that is based on the characteristics of the patient's gastrointestinal NET are needed.

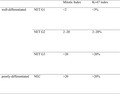

## 1 INTRODUCTION

### 1.1 Biology

Neuroendocrine tumors (NETs) are rare neoplasms, with an estimated annual incidence of 6.9/100 000. They arise from cells of the diffuse endocrine system,^1^, ^2^ which are mainly dispersed throughout the gastrointestinal (GI), pancreatic, and respiratory tracts.

Neuroendocrine cells are derived from either epithelial or neuroectodermal cells. GI‐NETs mainly arise from epithelial cells.^3^ NETs are positive by silver staining and express synaptophysin, neuron‐specific enolase, and chromogranin A. Many neuroendocrine cells contain membrane‐bound neurosecretory granules, which contain hormones and biogenic amines such as serotonin, corticotrophin, histamine, dopamine, substance P, neurotensin, prostaglandins, and kallikrein.^4^ The release of these substances into the systemic circulation leads to a variety of secretory syndromes, which can manifest as flushing, diarrhea, wheezing, rash, or even multiorgan failure.

Most NETs grow slowly over the years, and patients with NETs show signs and symptoms related to the mass of the tumor; however, about 30% of patients can have signs and symptoms associated with the hypersecretion of hormones from the tumor. Patients with nonresectable functional NETs who show signs and symptoms of hormonal hypersecretion can require systemic treatment for both control of the tumor and their signs and symptoms.^5^ NETs often secrete serotonin and other vasoactive substances, which lead to the typical carcinoid syndrome, characterized by flushing, diarrhea, and right‐sided valvular heart disease.

### 1.2 Epidemiology

A study based on the Surveillance, Epidemiology, and End Results (SEER) database showed that the age‐adjusted annual incidences of NETs arising from the appendix, cecum, duodenum, colon, stomach, jejunum/ileum, and rectum were 0.15, 0.16, 0.19, 0.20, 0.30, 0.67, and 0.86, respectively.^6^ Whereas Caucasian patients develop mainly midgut NETs, African American, Asian, and Native American patients more frequently develop rectal NETs. Female patients appear to develop NRTs of the stomach, appendix, or cecum more frequently, whereas NETs in male patients appear to be more frequent in the jejunum/ileum, duodenum, and rectum.^6^ Recent epidemiological inconsistencies have been reported for American and European countries vs Asian countries, where a higher incidence of rectal primaries has been noted.^7^, ^8^, ^9^ According to the SEER database, 53% of patients with NETs present with localized disease, 20% have locoregional disease, and 27% have distant metastases at the time of diagnosis.^1^ Individuals with a family history of NET in a first‐degree relative have a 3.6‐fold increased risk of disease.^10^ Thus far, no environmental risk factors have been conclusively identified. Analysis of NET survival rates from the SEER database found that the median overall survival (OS) for all NET patients was 9.3 years. Well‐differentiated tumors were found to have higher OS than moderately differentiated tumors (16.2 vs 8.3 years). The poorly differentiated, undifferentiated, and anaplastic tumors only had an OS of 10 months.^1^

### 1.3 Histologic staging

Whereas tumor grade, as measured by the mitotic rate and/or the Ki‐67 index, refers to the proliferative activity of neoplastic cells, differentiation refers to the extent to which tumor cells resemble their normal counterparts. Tumor grade is defined numerically, as follows: low‐grade (grade 1 [G1]) tumors have a mitotic rate from 0 to 1 per 10 high‐power fields (HPFs) or a Ki‐67 index from 0% to 2%; intermediate‐grade (G2) tumors have a mitotic rate from 2 to 20 per 10 HPFs or a Ki‐67 index from 3% to 20%; and high‐grade (G3) tumors have a mitotic rate >20 per 10 HPFs or a Ki‐67 index >20%.^11^ Note that tumor grade always should be measured in the most mitotically active areas of the pathology specimen, because a considerable degree of intratumor heterogeneity has been reported.^12^ According to the 2010 World Health Organization (WHO) classification, well‐differentiated NETs are subdivided into either G1 or G2 tumors, whereas poorly differentiated neuroendocrine carcinomas (NECs) were considered equivalent to G3 tumors. Because well‐differentiated, high‐grade NETs clearly exist (primarily in the pancreas), the WHO proposed a new classification in 2019 that distinguishes between well‐differentiated (low‐grade, intermediate‐grade, or high‐grade) GI/pancreatic NETs and poorly differentiated (high‐grade) GI/pancreatic NECs (Table 1).

**TABLE 1:** 2019 World Health Organization classification of GI neuroendocrine tumors (NETs)

		Mitotic Index	Ki‐67 index (%)
Well‐differentiated	NET G1	<2	<3
	NET G2	2‐20	2‐20
	NET G3	>20	>20
Poorly differentiated	NEC	>20	>20

Abbreviations: NEC, neuroendocrine carcinoma; NET, neuroendocrine tumor.

### 1.4 Diagnosis

**FIGURE 1:**
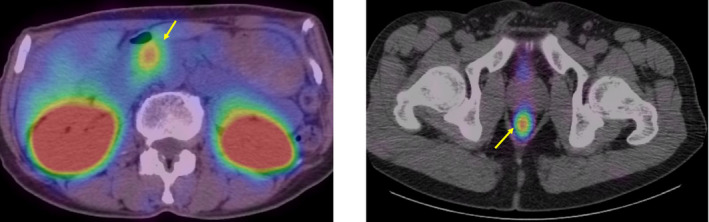
Abnormal accumulation at sites of the tumor seen on an indium‐111 (^111^In) pentetreotide somatostatin receptor scintigraphy scan (OctreoScan)

The diagnosis of NETs is based on histopathology, imaging, and clinical presentation. Computed tomography (CT) and magnetic resonance imaging (MRI) play a key role in assessing the location and extent of GI‐NETs. In a series of 64 patients with metastatic GI‐NETs, MRI was found to be more sensitive than CT for the detection of small liver metastases.^13^ Poorly differentiated NETs are commonly imaged by 18F‐fluorodeoxyglucose PET/CT. However, 18F‐fluorodeoxyglucose PET/CT is not recommended routinely, because 18F‐fluorodeoxyglucose PET/CT uptake can correlate negatively with the prognosis of well‐differentiated NETs.^8^ Functional imaging studies for patients with NETs are based primarily on tumor expression of somatostatin receptors, which is assessed by indium‐111 (^111^In) pentetreotide somatostatin receptor scintigraphy (SRS; OctreoScan, Figure 1).^14^ In patients with occult primary tumors, gallium 68 (^68^Ga) 1,4,7,10‐tetraazacyclododecane‐1,4,7,10‐tetraacetic acid (DOTA)–octreotate (DOTATATE) PET/CT scanning is useful for baseline whole‐body staging, the detection of small lymph node or bone metastases, and identification of the primary site.^15^, ^16^ Patients who present with signs and symptoms of carcinoid syndrome should receive testing of a 24‐hour urine sample for 5‐hydroxyindoleacetic acid (5‐HIAA), which is the breakdown product of serotonin.^17^ Additionally, a plasma 5‐HIAA assay has recently been reported to be as accurate as the 24‐hour urine 5‐HIAA measurement.^18^

## 2 SURGICAL TREATMENT FOR GI‐NETS

Surgery is the mainstay treatment of locoregional GI‐NET, which may be curative in the case of R0 resection. Patients with operable disease have better prognosis than those with inoperable disease, even in advanced stages.^19^, ^20^ The indications for the surgical treatment of patients with GI‐NETS are described in the European NET Society (ENETS) guidelines^21^ and the Japan NET Society (JNETS) guidelines.^22^ Endoscopic resection is an option for patients with early well‐differentiated GI‐NETs, which are thought to have a very low frequency of lymph node metastasis. Lesions which are technically difficult to resect endoscopically can be treated by local resection (partial resection without lymph node dissection). Lesions with possible lymph node metastasis can be treated by enterectomy with lymph node dissection. Recently, minimally invasive surgery (ex: laparoscopic or robotic‐assisted surgery) plays a relevant role in treatment of GI‐NET. Due to the rarity of NET, comparative data on minimally invasive surgery vs open surgery are scarce and the generation of new data on the basis of prospectively randomized trials is made more difficult. Consequently, many developments in minimally invasive therapy of NET are based on data and experience gained from other tumor entities and transferred to NET.^23^

### 2.1 Surgical treatment for gastric NETs

**FIGURE 2:**
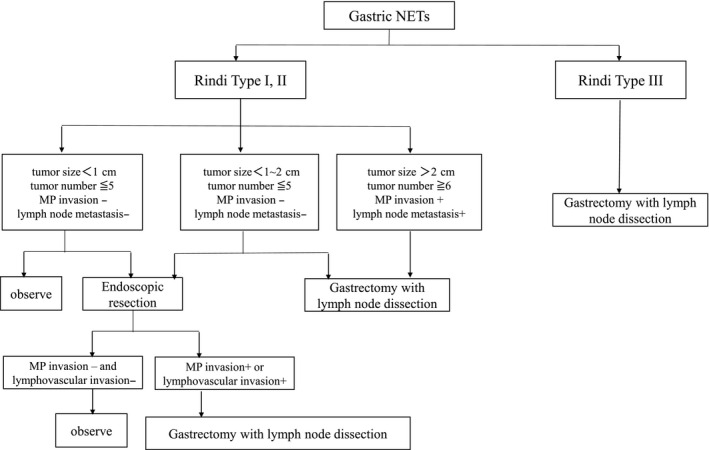
Algorism of surgical treatment for gastric neuroendocrine tumors

Gastrointestinal NETs arise from subepithelial, histamine‐secreting, enterochromaffin‐like cells. Type A gastritis is strongly associated with GI‐NETS. The recommendations for treatment suggest that the selected surgical procedure should be based on the Rindi classification (Table 2, Figure 2).^24^

**TABLE 2:** Rindi classification

	Type I	Type II	Type III
Characteristics of tumor	Multiple <1 cm	Multiple <1 cm	Solitary 1 cm with ulcer
Related disease	Atrophic gastritis Pernicious anemia	Zollinger‐Ellison syndrome MEN type I	
Pathology	well differentiated	well differentiated	well ~ moderately differentiated
Hypergastrinemia	+	+	−
Frequency (%)	70‐80	<5	15‐20

Abbreviation: MEN, multiple endocrine neoplasia.

Type I GI‐NETs occur in patients with chronic atrophic gastritis. They account for approximately 80% of gastric carcinoids.^25^ The chronic absence of gastric acid stimulates antral G cells to secrete excess serum gastrin, which, in turn, causes gastric neuroendocrine cell hyperplasia and the development of multifocal polypoid NETs. Type I NETs generally behave in a benign fashion, and aggressive treatment is rarely needed.

Type II gastric carcinoids are caused by hypergastrinemia in the setting of an underlying gastrinoma, primarily in patients with multiple endocrine neoplasia 1 (MEN1). Consequently, patients who have type II gastric NETs usually present with symptoms of Zollinger‐Ellison syndrome, such as diarrhea, heartburn, and peptic ulceration. Tumors tend to be small, multifocal, and relatively unaggressive. Elevated serum gastrin levels and low gastric pH are frequent in patients with type II gastric carcinoids. Management is conservative, and tumors might regress with successful treatment of the underlying gastrinoma.^26^ According to the JNETS guidelines, follow‐up or endoscopic resection is recommended for Rindi type I and II tumors with a diameter of 1 cm or less, the numbers of tumors are five or less, and are not invasive of the muscularis propria (MP).^22^ Antrectomy is also an option for patients with hypergastrinemia. However, gastrectomy with lymphadenectomy is recommended for cases with invasion of the MP, lymphovascular invasion, or suspected metastatic lymph nodes. Type III (or sporadic) gastric NETs occur in fewer than 15% of cases and are not associated with gastrin overproduction. Their malignant potential is considerably higher than that of type I and II tumors, and radical resection with lymphadenectomy is required.

The frequency of lymph node metastasis in GI‐NETs with tumor diameters of 1‐2 cm was reported to be 21%.^27^ Therefore, the JNETS guidelines recommend D2 dissection for GI‐NETs with tumors larger than 1 cm or invasion of the submucosal layer.^22^

### 2.2 Surgical treatment for duodenal NETs

Duodenal NETs often present as individual small, pale sessile lesions located mostly in the first or second part of the duodenum.^28^, ^29^ The recommended treatment for isolated NETs arising in the duodenum is endoscopic resection, if possible.^30^ Subcentimeter G1 duodenal NETs in nonampullary locations can be treated by endoscopic resection. Periampullary NETs and duodenal NETs larger than 2 cm should be considered for surgical resection. The treatment for duodenal NETs of between 1 and 2 cm remains controversial.^21^ Submucosal endoscopic dissection has a better rate of complete resection, but has a longer procedural time and higher complication rate (e.g., bleeding and perforation). Surgical treatment options include local excision with locoregional lymph node sampling or pancreatoduodenectomy (PD). In patients with suspected lymph node metastases, PD is the first choice for curative intent.

### 2.3 Surgical treatment for NETs of the small intestine

Most small bowel NETs originate in the distal ileum.^31^ Approximately 25% of patients have multifocal tumors at the time of diagnosis, which are often clustered in close proximity to each other.^32^ Although the malignant potential of intestinal NETs correlates with tumor size, even subcentimeter neoplasms can metastasize.^33^ The liver, mesentery, and peritoneum are frequent metastatic sites. Because the risk of metastasis to the lymph nodes is high, even when the tumor diameter is small,^34^ small bowel resection with dissection of the mesenteric lymph nodes should be performed.

### 2.4 Surgical treatment for NETs of the appendix

**FIGURE 3:**
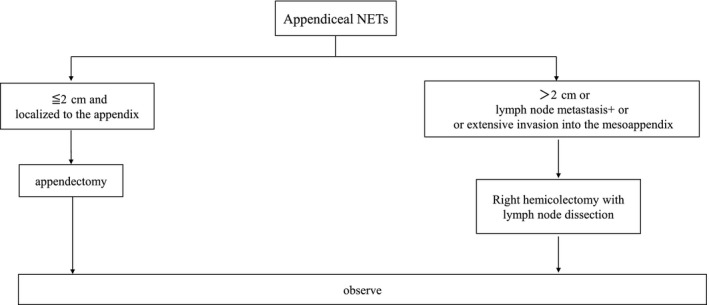
Algorism of surgical treatment for appendiceal neuroendocrine tumors

Neuroendocrine tumors of the appendix have been found in one of 300 appendectomy specimens, and most are found incidentally after surgery for appendicitis. The malignant potential of appendiceal NETs appears to be strictly related to tumor size. A study of a large series of patients did not find metastatic disease in 127 patients who had tumors smaller than 2 cm.^35^ Consequently, a simple appendectomy has been considered adequate for tumors smaller than 2 cm, whereas right hemicolectomy has been recommended for tumors larger than 2 cm. However, locoregional or distant metastases in patients with tumors measuring 1 or 2 cm in diameter have recently been reported.^36^ The negative prognostic factors for tumors of this size range include location at the base of the appendix, lymphovascular invasion, or extensive invasion of the mesoappendix (Figure 3).^36^ Therefore, right hemicolectomy with lymph node dissection is recommended for patients with invasive tumors. Rault‐Petit et al^37^ examined 403 patients with appendiceal NETs (26 patients with lymph node metastasis), but could not identify the predictors of lymph node metastasis, and the predictors remain unknown.

### 2.5 Surgical treatment for NETs of the colon and rectum

Colonic NETs most frequently arise in the ascending colon. Colectomy with regional lymphadenectomy has been recommended for these tumors.^38^ Colonic NETs distal to the cecum tend to be more aggressive than rectal NETs and are often poorly differentiated.^39^

**FIGURE 4:**
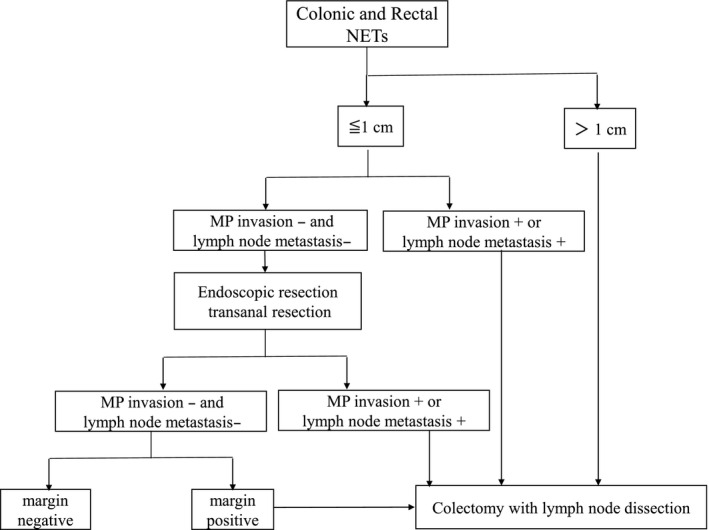
Algorism of surgical treatment for colonic and rectal neuroendocrine tumors

Rectal NETS with a tumor diameter greater than 2 cm show a very high frequency of lymph node metastasis (58%‐76%), and therefore these tumors are indications for rectal resection plus lymph node dissection.^40^, ^41^ On the other hand, for tumors with a diameter of 1‐2 cm, ENETS guidelines recommend local resection if neither MP invasion nor lymph node metastasis is suspected.^42^ The reported predictors of lymph node metastasis for rectal NETs include the following characteristics: (a) tumor diameter >1 cm; (b) ulcerations; (c) presence of vascular invasion.^40^, ^41^ In Japan, the frequency of lymph node metastasis in NETs of the rectum ranges as high as 18.5%‐30.4%, even for tumors of 1‐2 cm. Therefore, according to the JNETS guidelines, patients with tumors (a) larger than 1 cm, (b) MP‐invasive, or (c) suspected to have lymph node metastases should undergo surgical resection with lymph node dissection.^22^ We recommend that patients with tumor diameters of 1 cm or smaller and MP invasion or without suspicion of lymph node metastasis should first undergo local resection (endoscopic resection or transanal resection); and if a histopathological examination identifies invasion of the MP, vascular invasion, or positive surgical margins, rectal resection with lymph node dissection should be performed (Figure 4).

### 2.6 Chemotherapy and surgical treatment for NETs associated with metastases

In recent years, the therapeutic armamentarium for metastatic GI‐NETs has expanded considerably. New systemic treatments for tumor and syndrome control have been shown to delay progression as well as diminish symptoms related to hormone secretion. Everolimus is an inhibitor of the mammalian target of rapamycin (mTOR). The RADIANT‐4 trial showed that everolimus significantly improved PFS over placebo in advanced well‐differentiated, nonfunctional lung or GI‐NETs.^43^ Bevacizumab is a vascular endothelial growth factor (VEGF) inhibitor, which was shown in the BETTER trial to be safe and potentially efficacious in progressive, metastatic, well‐differentiated GI‐NETs.^44^ Combined therapy yielded improved response rates and median survival rates.^45^ A more recent phase II/III study comparing outcomes between combined therapy of 5‐fluorouracil with doxycycline or streptozocin found that there is improved survival when using a combined therapy of 5‐fluorouracil and streptozocin compared with 5‐fluorouracil with doxycycline.^46^

Moreover, since cytoreduction surgery for NETs with metastatic lesions can control hormonal hypersecretion and alleviate symptoms, cytoreduction surgery is recommended.^21^ However, the surgical resection of multiple organs for NETs associated with metastatic lesions is highly invasive, and a careful assessment of the indications for surgery and the risks and benefits of surgical resection is required.

For patients with liver metastases, the 5‐year survival rate after hepatectomy has been reported to range from 61% to 81%, and rates of improvement of associated signs and symptoms, including hormonal symptoms, has been reported to range from 90% to 100%.^47^, ^48^, ^49^, ^50^, ^51^ Cytoreduction surgery is often recommended if at least 90% of the liver tumors can be resected.^52^, ^53^, ^54^, ^55^ Various intraoperative or percutaneous ablative procedures, including alcohol ablation, cryoablation, and radiofrequency ablation, are also commonly performed.^56^, ^57^ A nonrandomized study investigated liver transplantation vs no transplantation in 88 patients who met strict eligibility criteria for transplantation. The transplantation patients obtained significant survival, with a 10‐year OS rate of 51% in the transplantation arm vs 22% in the nontransplantation arm (*P* = .001).^58^ Cytoreduction surgery for patients with lung metastases and peritoneal dissemination might also reduce the signs and symptoms associated with these types of metastases.^59^, ^60^

The resection of primary tumor has been recently reported to possibly lead to the prolonged survival of patients with GI‐NETs associated with metastases.^61^ Additional variables related to survival for each NET subtype were identified and might help select patients who benefit from removal of the primary tumor.

## 3 CONCLUSIONS

In general, surgery is the first‐line treatment for patients with localized GI‐NETs, and it should also be considered for palliation in patients with metastatic or bulky disease. The indications for surgery vary, based on the organ where the NET arose; therefore, an understanding of the patient's clinical state and individualized treatment that is based on the characteristics of the patient's GI‐NET are needed.

## DISCLOSURE

Conflicts of Interest: The authors declare no conflicts of interest.

Author Contribution: Study conception and design – K. Eto, N. Yoshida, S. Iwagami, and H. Baba; acquisition of the data – K. Eto, N. Yoshida, S. Iwagami; writing of the manuscript – K. Eto, N. Yoshida, S. Iwagami, and H. Baba. All authors approved the final manuscript.
